# Evaluation of predominant risk factors for type 2 diabetes mellitus among out-patients in two Nigerian secondary health facilities

**DOI:** 10.4314/ahs.v21i2.27

**Published:** 2021-06

**Authors:** Chinonyerem O Iheanacho, Doyin O Osoba, Uchenna IH Eze

**Affiliations:** 1 Department of Clinical Pharmacy and Public Health, Faculty of Pharmacy, University of Calabar, Nigeria; 2 Department of Clinical Pharmacy and Biopharmacy, Faculty of Pharmacy, Olabisi Onabanjo University, Sagamu, Nigeria

**Keywords:** Evaluation, type 2 diabetes, risk factors, Nigeria, primary prevention

## Abstract

**Background:**

Prevention of type 2 diabetes is enabled by identification and effective management of risk factors.

**Objectives:**

To evaluate the predominant risks for type 2 diabetes and identify persons at highest risk in a population; to facilitate the understanding of implications for practice.

**Methods:**

Cross-sectional survey using Canadian diabetes risk assessment questionnaire was conducted among non-diabetic persons who visited two secondary hospitals. SPSS version 18 was used for data analysis.

**Results:**

A total of 300 respondents participated in the study, with 25.7% having family history of type 2 diabetes, while 160 (53.3%) were at high risk of developing the disease. Males (62.5%), overweight (65.1%) and obese (82.6%) participants, were at higher risk. Others found to be at high risk were respondents with high waist circumference (55.6%), respondents who did not exercise (77.0%), those who did not eat fruits/vegetable daily (54.4%), those with high blood pressure (67.5%) and those who have had raised blood sugar in the past (71.0%).

**Conclusion:**

Majority of the study participants was at high risk for type 2 diabetes, male participants had higher risks and lifestyles/habits were the major risks for developing the disease..

## Introduction

Chronic diseases including diabetes have been major determinants of quality of life and have created high disease burden in several people. Incidence of diabetes is seen to be on rapid increase in low and middle income countries and is a major cause of kidney failure, lower limb amputation, blindness and some cardiovascular events and also the seventh leading cause of death in 2016^1^.

Type 2 diabetes mellitus is a major cause of morbidity and mortality rate^2^,^3^ and this usually results from micro-vascular and macro vascular complications affecting multiple organ systems. People with diabetes have a greatly increased risk of nephropathy^2^,^4^, blindness, myocardial infarction, stroke, necessary limb amputation, and a host of other maladies. The onset and progression of these complications are strongly linked to the presence of sustained hyperglycemia. The complication rate and the severity of complications increase as the duration of diabetes increases.^4^ Co-morbidities such as hypertension and dyslipidemia increase the risk for micro-vascular and macro-vascular complications and mortality^4^,^3^.

Several risk factors for type 2 diabetes have been identified, among which are family history, overweight, obesity, smoking, hypertension^5^, history of gestational diabetes, poor nutrition during pregnancy, ethnicity and physical inactivity 6. Other factors have been associated with the emergence of diabetes mellitus in the general population, among which are age, education, income, gender and family history 7,8. It is hence a metabolic disorder with multiple etiologies and results from insulin defect or deficiency 5. Management of diabetes risk factors aids in the prevention and delay of on-set of the disease and its complications 8.

Diet, exercise, weight control, and medications are the mainstays of diabetic care 9. Obesity is very common in type 2 diabetes and contributes greatly to insulin resistance. Weight reduction and exercise improve tissue sensitivity to insulin and allow its proper use by target tissues 9. Educational interventions on lifestyle modification, based on the risks of individuals will effectively enhance the primary prevention of this disease among people at risk.

Nigeria is said to be undergoing epidemiological transition where double burden of infectious diseases and non-communicable diseases is seen^10^, and this is mainly attributed to lifestyle changes. It is therefore, necessary to evaluate the prevailing predisposing lifestyles for the risk of type 2 diabetes in the population. The risk of diabetes mellitus and its complications has been associated with increasing age, hence this study is focused on patients who are 45 years and above.

Type 2 diabetes mellitus is rapidly on the increase among the Nigerian population and this is not merely a result of family history, but majorly lifestyle-related. This study aimed to evaluate the factors associated with higher risks for type 2 diabetes and persons with highest risk; among patients visiting the general outpatients' department in the secondary health facilities, for effective and targeted primary prevention approach. This will also facilitate the understanding of implications for practice, in terms of those at highest risk.

## Methods

This study was conducted in the out – patients' department (OPD) of two secondary health institutions in Ogun State. A cross - sectional survey was conducted to assess diabetes risk with the use of the Canadian diabetes risk assessment questionnaire (CANRISK) 11, which was adapted to suit our respondents. The questionnaire was divided into two sections.

Section A obtained socio-demographic information including reasons for visiting the hospital. Section B consisted of 20 questions according to Canadian diabetes risk assessment questionnaire (CANRISK), arranged in an ordinal format of yes, no, don't know, everyday and not every day, which was analysed by the addition of the points 11.

The questionnaire was assessed for validity by a clinical pharmacist and a medical doctor who specialized in endocrinology. It was also pre-tested among thirty patients of similar demographics as the study population. The sample size ″n_0_″ was determined using Cochran formula and 384 respondents were included in the study.^12^ Non diabetic patients of 45 years old and above were included in the study. Simple random sampling was used to collect data over a 2-month period from patients in the out-patients' clinics. The tool was self-completed, however, reading assistance was provided for respondents who could not read. Measurement of the height, weight and waist circumference of each patient were taken and recorded.

Data were inputed into Microsoft excel, while SPSS version 18 was used for further analysis and results were presented as simple percentages and proportions.

Section B of the questionnaire was analysed thus:

- Age 40–44.....0 point, 45–54....7 points, 55–64....13points, 65–74...15points
- Gender Male ........6 points, Female........0 point
- BMI <25...0 point, 25–30....4 point, 30–34....9 points, >35......14 points
- Waist circumference
  Male: 94cm.....0 point, 94–102 cm.......4points, >102 cm......6 points
  Female: 80cm.....0 point, 80 – 88cm, 4 points, >88 cm....6 points
- Physical activity, yes....0 point, No....1points
- Eating of vegetable /fruits, everyday....0 point, Not every day.....2 points
- Tested with high blood pressure, yes...4 points, No....0 point
- Tested with high blood glucose, yes.....14points, No ...0 point
- Macrocosmic baby, yes.....1 point, no .....0 point
- Family history:
  Yes ....2 points
  “No or Don't know” for everyone....0 point
- Educational level: < secondary school leaving certificate......5 points
  >Secondary school leaving certificate......0 point

Addition of points from question 1 to 11 with the age will help to estimates the risk of developing type 2 diabetes in the next 10 years.

Estimated 10 year risk of developing type 2 diabetes is as follows;

- < 21---- Low risk
- 21 - 32----Moderate risk
- > 32----High risk

After interpreting these data using SPSS, the patients were classified into three (3) groups: low risk, moderate risk or high risk group, using the Canadian diabetes risk assessment questionnaire (CANRISK).

### Ethical approval

Ethics approvals were obtained from the hospitals' managements with reference numbers SHI.58/VOL.1/83 and IT/I/VOL1 for Isara and Ijebu Ode General Hospitals respectively. Informed consent was also obtained from the respondents prior to the study. Confidentiality and anonymity of the patient's information were maintained during and after the study.

## Results

From a total of 384 patients who were recruited for the study, 300 participated; resulting in a response rate of 78.1 %. The percentage of participants from Ijebu Ode General Hospital was 66.7% and 33.3% were from Isara Remo General Hospital.

From the total of 300 respondents, a total of 80 (26.7%) were within the age bracket of 45 and 54 years, 204 (68.0%) were females, 225 (85.0%) were married, 183 (61.0%) were Christians, 90 (30.0%) had only primary education, 262 (87.3%) were of the Yoruba tribe, and 137 (45.7%) had an average monthly income of less than 5,000 Naira (12 U.S Dollars). The female to male ratio in this study was 1: 0.5.

From the screening conducted, 152 (50.7%) of the patients were within the BMI range of 25.0 to 29.9, which is the range of overweight, and 40 (13.3%) had the normal waist circumference (31 to 35 inches). Average weight of the patients was 70.17, height was 1.67, average body mass index was 26.22, and average waist circumference was 38.89. One hundred and sixty one (53.7%) of the patients had their meals three (3) times daily, 171 (57.0%) ate snacks in between meals while 78 (26.0%) took it less than once a week. It was also observed that 173(57.7%) of the patients did get involved in some form of exercise resulting from the nature of their job. Two hundred and twenty six (75.3%) of the respondents did not eat fruits and vegetables daily. See Table 1 for details.

**Table 1:** Modifiable risks for type 2 diabetes mellitus in respondents

Variables	Frequency (n)	Percentage (%)
**Body Mass Index (BMI)**		
Underweight	14	4.7
Normal	111	37.0
Overweight	152	50.7
Obese	23	7.7
**Waist Circumference**		
Low	9	3.0
Normal	40	13.3
Moderate	175	58.3
Large	53	17.7
Very large	23	7.7
**Respondents' meal** **pattern**		
**Meals per day**		
Once	3	1.0
Twice	36	12.0
Thrice	161	53.7
More than 3	100	33.3
**Snacks**		
Yes	171	57.0
No	129	43.0
**Frequency of snacks**		
Daily	14	4.7
2 – 3 times weekly	38	12.7
Once a week	58	19.3
Less than once a week	78	26.0
No response	112	37.3
**Exercise**		
Yes	173	57.7
No	127	42.3
**Ate fruits and** **Vegetables**		
Everyday	74	24.7
Weekly	165	55
Monthly	46	15.3
None	15	5.0

Results also show that 160 (53.3%) of the respondents had high blood pressure, 140 (46.7%) were not aware of their blood pressure, 252 (84.0%) reported normal range of cholesterol, 223 (74.3%) did not have a family history of diabetes and 227 (75.5%) did not have macrocosmic baby (high weight baby, at birth). See Table 2.

**Table 2:** Occurrence of co-morbidities and inherent risk factors in respondents

Co-morbidities	Frequency n = 300	Percentage (%)
**Patients with high blood pressure**		
Yes	160	53.3
No	140	46.7
**Patients with either high, normal or don't** **know their blood pressure**		
High	140	46.7
Normal	20	6.6
Don't know	140	46.7
**Patients with high cholesterol**		
Yes	28	9.3
No	252	84.0
Don't know	20	6.7
**Previously tested high blood sugar**		
Yes	41	13.7
No	259	86.3
**Family history of diabetes**		
Yes	77	25.7
No	223	74.3
Specification		
No family history of diabetes	223	74.3
Mother	20	6.7
Father	19	6.3
brother /sister	27	9.0
Children	4	1.3
Other family member	7	2.3
**Macrocosmic baby**		
Yes	73	24.3
No	227	75.7

**Figure 1:**
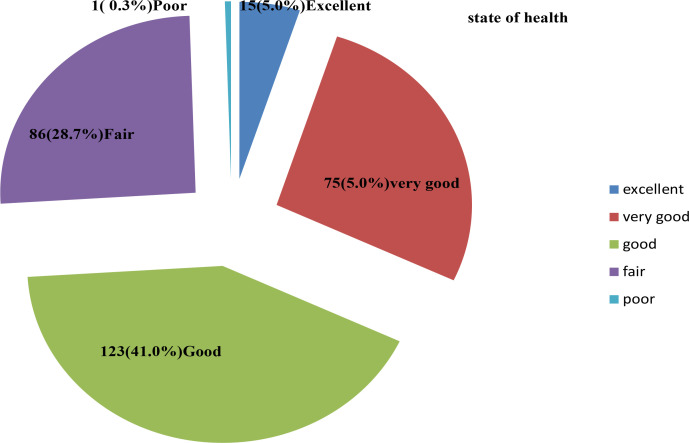
Respondents' State of health

From the 300 questionnaires administered 123(41.0%) patients state of health were good and others are as shown in Figure 1.

Following the risk evaluation, 62.5% of the males were at high risk of developing type 2 diabetes mellitus, 61.5% of participants with less than tertiary education were at high risk. A total of 65.1% and 82.6% of the overweight and the obese participants respectively were also at high risk. Similarly, 55.6% of respondents with higher than normal waist circumference were at high risk, 77.0% of respondents who were not involved in exercise were at high risk, 54.4% of those who did not eat fruits and vegetable daily were at high risk, while 67.5% of those with high blood pressure and 71.0% of respondents with a previous history of high blood sugar respectively, were at high risk. Family history of diabetes (14.3%) and birth of macrocosmic baby (14.9%) did not pose high risk to the development of type 2 diabetes mellitus respectively. Other data are as shown in Table 3.

**Table 3:** Shows variables and their rate of risk of diabetes mellitus. n= 300

s/n	Items	Variables	Frequency	Percentage%	High risk%	Moderate risk %	Low risk %
1.	Gender	Male	96	32.0	62.5	25	12.5
2.	Education	Equal or below secondary school level	208	69.3	61.5	26.0	12.5
3.	BMI	Overweight	152	50.7	65.1	26.3	8.3
		Obesity	23	7.7	82.6	17.3	0
4.	Waist Circumference		251	87.0	55.6	28.7	15.7
5.	Exercise	Respondent that did not do any exercise	127	42.3	60.3	23.6	15.7
6.	Fruits & vegetable	Respondent that did not eat fruits & vegetable daily	226	75.3	54.4	28.3	17.25
7.	Blood pressure	Respondent with high blood pressure	160	53.3	67.5	25.6	6.9
8.	High blood sugar	Respondent that had been tested with high blood sugar in the past	41	13.7	71.0	20.0	9.0
9.	Macrocosmic baby	Respondent that had given birth to baby weighing over 4.0kg	69	23.0	21.6	44.9	33.5
10.	Family history	Respondents with relatives who had diabetes	77	25.7	40.3	33.8	26.0

Association between high blood pressure, body mass index, family history of diabetes; and appearance of high blood sugar during an illness or during pregnancy, were very significant (p= 0.001), (p=0.0029) and (p=0.000) respectively.

**Figure 2:**
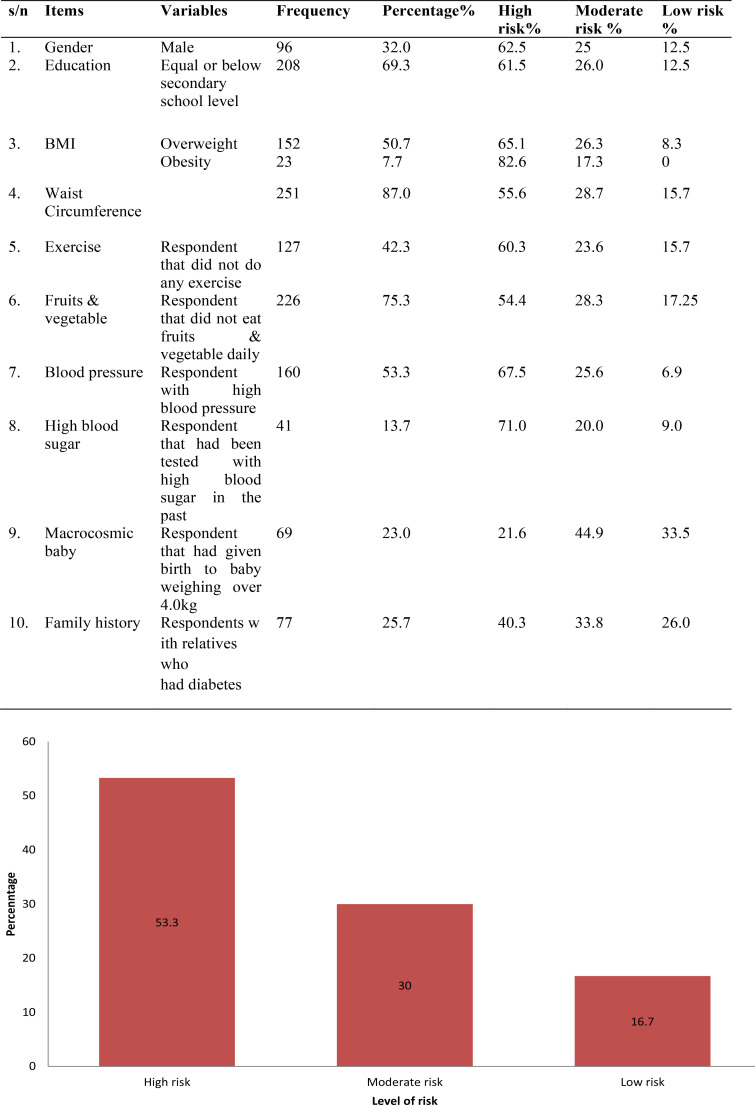
Respondents level of Risk in accordance with CANRISK

Also, 160 (53.3%) of the total population were at high risk. Figure 2 shows the level of risk accordance with CANRISK.

## Discussion

Majority of the study participants were females, married and also middle aged. Majority also had low educational background (primary education) and had low monthly income. The population is hence, seen to have low socio-economic background. A similar study also comprised mostly of respondents of similar age range, but who had high school education 13. Socio-demographics have been identified in previous studies as a major determinant of the risks for developing type 2 diabetes mellitus 5,8. It is therefore, an important variable in the assessment of diabetic risk factors.

Findings in this study show that males and respondents who had lower educational qualifications were at high risk of developing type 2 diabetes. Another study in Saudi Arabia also reported lower educational qualification to be significantly associated with diabetes mellitus and males were also reported to be more at risk than the females 8. In another study, females were seen to have a higher risk of developing type 2 diabetes than their male counterparts 7. Varied lifestyle between the males and females increases their susceptibility to disease conditions.

Also, half of the respondents were overweight and only few of them had waist circumference within the normal range. High risk for diabetes was found in participants who were overweight, obese and had high waist circumference. Body Mass Index (BMI) was also found to be significantly associated with mellitus and singly presented the highest risk for the disease. This is consistent with another study which reports overweight, BMI and obesity as factors that raise the chances of developing type 2 mellitus 14. According to a study in the United States, the widespread and high prevalence of type 2 diabetes is associated with increasing rate of overweight and obesity in the population 15. Exercise is recognized as a means of achieving weight loss in overweight or obesity 16, hence should be encouraged, with relevant assessments in the population.

Although, majority of the study participants did not have a family history of diabetes, this variable was significantly associated with the development of raised blood sugar. Similarly, family history was reported to be significantly associated with type 2 diabetes in previous studies 5, 14. Having a first degree relative with type 2 diabetes mellitus increases the risk of developing the disease 6. Although, this is a non-modifiable risk factor for diabetes, the disease development could be delayed by lifestyle choices. Educational intervention on lifestyle choices is therefore very relevant in this groups of persons.

Furthermore, many of the study participants did not have three score meals and also did not take fruits and vegetables daily. Meanwhile, more than half of those who took snacks took it between meals. The poor nutrition observed in this study may be related to the respondents' low socio-economic background. Poor nutrition has been identified as a risk factor for diabetes mellitus 6. Participants who did not eat fruits daily were also observed to be at high risk. Healthy diet is one of the practices that delays or prevents the development of diabetes 1.

Also, more than half on the respondents were involved in exercises and the study observed that those who were not involved in exercise were at high risk of developing type 2 diabetes. Another study showed that less than half of the study population did not engage in regular exercise 17. Physical activity is very essential in the management of high blood sugar as well as the general health of persons who live with diabetes mellitus and pre-diabetes 9. It aids in the reduction of cardiovascular risk factors and improves quality of life 9. A study indicates the importance and usefulness of aerobic and resistance exercise in the control of type 2 DM 18. Also, regular exercise is described as one of the ways to delay or prevent diabetes mellitus 1.

The study showed that more than half of the study population had high blood pressure; a condition that was also seen to be significantly associated with the development of high blood sugar in the study. A similar study reports low prevalence of hypertension among its study population 7. High blood pressure has been listed by the International Diabetes Federation as a risk factor for diabetes 6, therefore, interventions for primary prevention of type 2 diabetes mellitus should also be targeted at hypertensive patients.

Also, those who previously tested positive to hyperglycemia during illness or pregnancy were observed to be at high risk of developing type 2 DM. This finding is consistent with another study were it was reported that hyperglycemia tests during pregnancy, predicts an increased risk of glucose intolerance after pregnancy 19. Previous diagnosis of gestational diabetes increases the risk of developing diabetes mellitus 6. Therefore, regular screening allows for early detection and secondary prevention of the disease 8.

Findings from the study also show that high risk for type 2 diabetes mellitus was found in majority of the respondents, while those with low risk were the least in population. Although, the emergence of type 2 diabetes is seen to be on the rise, an increased upsurge may be seen, following findings from this study. This emphasises the need for diabetes education targeted at out-patients who visit the hospitals for non-diabetes-related reasons.

The study findings from the study appear to suggest that males, persons of lower educational background, who were over-weight or obese, persons with higher waist circumference and without regular exercise; were at highest risk of developing type 2 diabetes mellitus. Lifestyle modification is therefore, the most effective approach towards the prevention of the disease in this group of persons. Healthcare experts-facilitated diabetes prevention programs through educational interventions; with focus on this group of persons is also essential in practice. The identification and development of appropriate preventive measures have become imperative, following life-threatening complications of the disease, treatment costs and treatment failures^20^. This approach will facilitate the primary prevention of type 2 diabetes. Meanwhile, generalisation of the findings from the study is limited by its inclusion of only outpatients.

## Conclusion

Majority of the study participants were at high risk of developing type 2 diabetes mellitus, while only few had low risks for the disease. Male participants had higher risks than their female counterparts. Although the risks were related to the lifestyles and family history of the respondents, it was predominantly lifestyle-related. Other socio-demographics such as low educational qualifications and overweight/obesity were also major risks for type 2 diabetes mellitus among the study participants. This emphasises the relevance of routine diabetes education with focus on lifestyle choices, even among persons with no family history of diabetes.
